# Supplementary material for: Chromothripsis during telomere crisis is independent of NHEJ, and consistent with a replicative origin
Source: Genome Res. 2019 May;29(5):737–49. doi: 10.1101/gr.240705.118 (PMC6499312; doi:10.1101/gr.240705.118)
Supplement: Supplemental Material [file supp_gr.240705.118_Supplemental_file_1.zip › contigs/annotated_contigs/DB53/contig.2.DB53_length_723_mean_cov_5.54910096819.docx]

**DB53_length_723_mean_cov_5.54910096819**

```
ACTCTCTGAGGCCCCTTCCCTCAGCTGTGACTTGGGACAAACCATACCTATCTCACAGGGCTATTGTGAGGCCAACGCAAATCTGCAGG
 >chr8:134772055-134772314 + E=5e-144
GGATTCTCAGCTCAAGAGGAAGAACACAGCAATTCGCAAATGGCAGCTGTTATTAGTTTACATTTAAATCTAGTTTTGCTGATTTGACA

TGTTCACTTGTTAGTTTTAAAAATAGCCTTCAGGCCGGGGGCGATGGCTCACACCTGTAATCCCAGCACTTTGGGAGGCCA|TG|ATGG
                                                                                    >ch
CAGAAGGTGAAGGGGAAGCAAGGCATCTTCACAAGGTGGCTGGAGGGAGAATGAACAGGAGGAACTACCAAACACTTTTAAAACCATCA
r8:132348253-132348713 + E=4e-251
GATCTCCTGAGAACTCACTTACTATAATGAGAACAGCATGGGGAAGACCACACCCGTGATTCAATTACTTCCACCTGGTCTCTCCCCTG

ACTCTCGGGATTATCAGGGTTATGGGGCTTACAACTGAAGATGAGATTTTGGGTGAGGACACAGCCAAACCATATCACCAACACTTTAC

CCTGATACTGGTTCCGACAAGACTGTTCTTTGTACTACAGTGTAATAATACCAATAATTATAGCCAAAGTTTAGCCGAACACTTATTAT

GAGCCAGACATATATTAAATGTATGTGTATTAACAATTCGAGATAGGTATTACTATTTTTCCACTTTATTAGTGAGAAGCACGAGGCAC

AGTGACTGAATTA
```
